# Low red/far-red ratio as a signal promotes carbon assimilation of soybean seedlings by increasing the photosynthetic capacity

**DOI:** 10.1186/s12870-020-02352-0

**Published:** 2020-04-08

**Authors:** Feng Yang, Qinlin Liu, Yajiao Cheng, Lingyang Feng, Xiaoling Wu, Yuanfang Fan, Muhammad Ali Raza, Xiaochun Wang, Taiwen Yong, Weiguo Liu, Jiang Liu, Junbo Du, Kai Shu, Wenyu Yang

**Affiliations:** 1 College of Agronomy, Sichuan Agricultural University, Huimin Road 211, Wenjiang District, Chengdu, 611130 People’s Republic of China; 2 Sichuan Engineering Research Center for Crop Strip Intercropping System, Chengdu, 611130 People’s Republic of China; 3 Key Laboratory of Crop Ecophysiology and Farming System in Southwest, Ministry of Agriculture, Chengdu, 611130 People’s Republic of China

**Keywords:** Light quality, Intercropping, Soybean, Shade, Photosynthesis, CO_2_ assimilation

## Abstract

**Background:**

Shading includes low light intensity and varying quality. However, a low red/far-red (R/Fr) ratio of light is a signal that affects plant growth in intercropping and close- planting systems. Thus, the low R/Fr ratio uncoupling from shading conditions was assessed to identify the effect of light quality on photosynthesis and CO_2_ assimilation. Soybean plants were grown in a growth chamber with natural solar radiation under four treatments, that is, normal (N, sunlight), N + Fr, Low (L) + Fr, and L light.

**Results:**

Low R/Fr ratio significantly increased the total biomass, leaf area, starch and sucrose contents, chlorophyll content, net photosynthetic rate, and quantum efficiency of the photosystem II compared with normal R/Fr ratio under the same light level (*P* < 0.05). Proteomic analysis of soybean leaves under different treatments was performed to quantify the changes in photosynthesis and CO_2_ assimilation in the chloroplast. Among the 7834 proteins quantified, 12 showed a > 1.3-fold change in abundance, of which 1 was related to porphyrin and chlorophyll metabolism, 2 were involved in photosystem I (PS I), 4 were associated with PS II, 3 proteins participated in photosynthetic electron transport, and 2 were involved in starch and sucrose metabolism. The dynamic change in these proteins indicates that photosynthesis and CO_2_ assimilation were maintained in the L treatment by up-regulating the component protein levels compared with those in N treatment. Although low R/Fr ratio increased the photosynthetic CO_2_ assimilation parameters, the differences in most protein expression levels in N + Fr and L + Fr treatments compared with those in N treatment were insignificant. Similar trends were found in gene expression through quantitative reverse transcription polymerase chain reaction excluding the gene expression of sucrose synthase possible because light environment is one of the factors affecting carbon assimilation.

**Conclusions:**

Low R/Fr ratio (high Fr light) can increase the photosynthetic CO_2_ assimilation in the same light intensity by improving the photosynthetic efficiency of the photosystems.

## Background

Light is an essential factor for crop growth and development in agricultural production [1]. Intra- or interspecies plant mutual shading often affects crop light interception [2], especially in intercropping and close planting system which are important cultivation methods in increasing resource utilization and yield [3]. Plants can perceive shading that enable them to acclimate and adjust their phenotypic and physiological characteristics to compete for limited light resource [4]. Shade often leads to elongation responses in the stem, petioles, and leaves in shade-sensitive plant species [5].

Plant shading reduces the amount of photosynthetically active radiation (PAR) and changes the spectral composition of light [6, 7]. Plants selectively absorb red (R) wavelengths through photosynthetic pigments. The far-red (Fr) spectrum is relatively enriched due to radiation reflected and transmitted by the green leaves of neighboring plants [8]. A resulting decrease in R/Fr ratio in the surrounding environment is observed. The changes in light intensity and quality under shade differ from those under low light condition [9, 10]. Low light conditions achieved using black nylon nets or fabrics do not alter the spectral composition of light, particularly the R/Fr ratio [11, 12].

Phytochromes play a key role in the perception of the R/Fr ratio signal and regulation of the plant photomorphogenesis via gene expression and physiological processes [1]. Phytochromes exist in two forms, that is, R light absorbing Pr and Fr light absorbing Pfr [1, 5]. The equilibrium between these two forms dynamically changes with the change in composition of the light spectrum within the 300–800 nm range [13]. A low R/Fr ratio is an important signal factor in shade avoidance [14]. Responses to low R/Fr ratio include increased stem elongation, decreased leaf area and branching, and changes in chlorophyll (Chl) content [8, 15, 16]. Low R/Fr ratio in normal or low light condition significantly increases the soybean biomass (dry weight) compared with normal R/Fr ratio (approximately 1.2) [6, 9].

A low R/Fr ratio indicates high enrichment of the Fr light spectrum in the plant canopy. Plant leaves absorb less amount of Fr light (λ > 700 nm); therefore, it contributes less towards the quantum yield of photosynthesis [17]. However, Zhen and van Iersel [18] reported that Fr light is required for efficient photochemistry and photosynthesis. Similarly, the Fr light of the shade is higher than that of low light, thereby increasing the net photosynthesis rate (*P*_n_) under the same light intensity condition [11], resulting in increased the whole-plant net assimilation [6, 9]. Typically, a shorter wavelength is used with longer wavelengths to enhance its photosynthetic efficiency, and this overall phenomenon is called the Emerson enhancement effect [18]. The reverse effect, which is the enhancement of the quantum yield of short light wavelength by Fr light (e.g., at low R/Fr ratio) in different light intensity environments, has not received considerable attention. Some studies only indicate that Fr light can increase plant photosynthesis and biomass [6, 18]. Therefore, in uncoupling R/Fr ratio from shade, whether low R/Fr ratio (Fr light enrichment) in different light intensity regulates the photomorphogenic process needs further analysis.

Photosystems I and II (PS I and II, respectively) operate in series to carry out the primary photochemical reactions of photosynthesis [18]. These processes are involved in light absorption and energy and electron transfer, which are carried out by different related proteins [11, 19]. Proteomic analysis has been used to identify changes in plant photosynthetic proteins in salt stress [20], water deficit [21], low phosphate [22], and chlorophyll deficient [23]. We previously investigated the response of soybean photosynthetic proteins to shade condition by using isobaric tagging for the relative and absolute quantification (iTRAQ) approach [11]. However, to our best knowledge, the proteomic analysis of photosynthesis in uncoupling light intensity and R/Fr ratio from shade is important to determine the R/Fr ratio regulating the photosystems related-proteins, which affect photosynthesis and CO_2_ assimilation.

Soybean (*Glycine max* (L.) Merr.) is one of the most important cultivated crops of protein and oil worldwide [24, 25], which is often used for rotation or to intercrop with other crops because it can fix atmospheric nitrogen [3]. However, soybean often suffer from intra- or interspecies mutual shading [9]. Shade increases the height but decreases the biomass, chlorophyll contents, and photosynthesis of soybean plant [2, 26]. Likewise, we previously reported the response of photosynthetic proteins to shade and low light by using iTRAQ-quantitative proteomic analysis, and found that Fr light enrichment of shade increases *P*_n_ by up-regulating the gene expression levels of differential proteins compared with low light [11]. Thus, low R/Fr ratio may be a signal in promoting CO_2_ assimilation by increasing the photosynthetic capacity. Therefore, this work aims to analyze the soybean morphology, carbohydrate, and photosynthesis in response to low R/Fr ratio in different light intensities and reveal the effect of low R/Fr ratio on soybean photosynthesis using the iTRAQ technique in different light intensities.

## Results

### Morphological characteristics

**Fig. 1:**
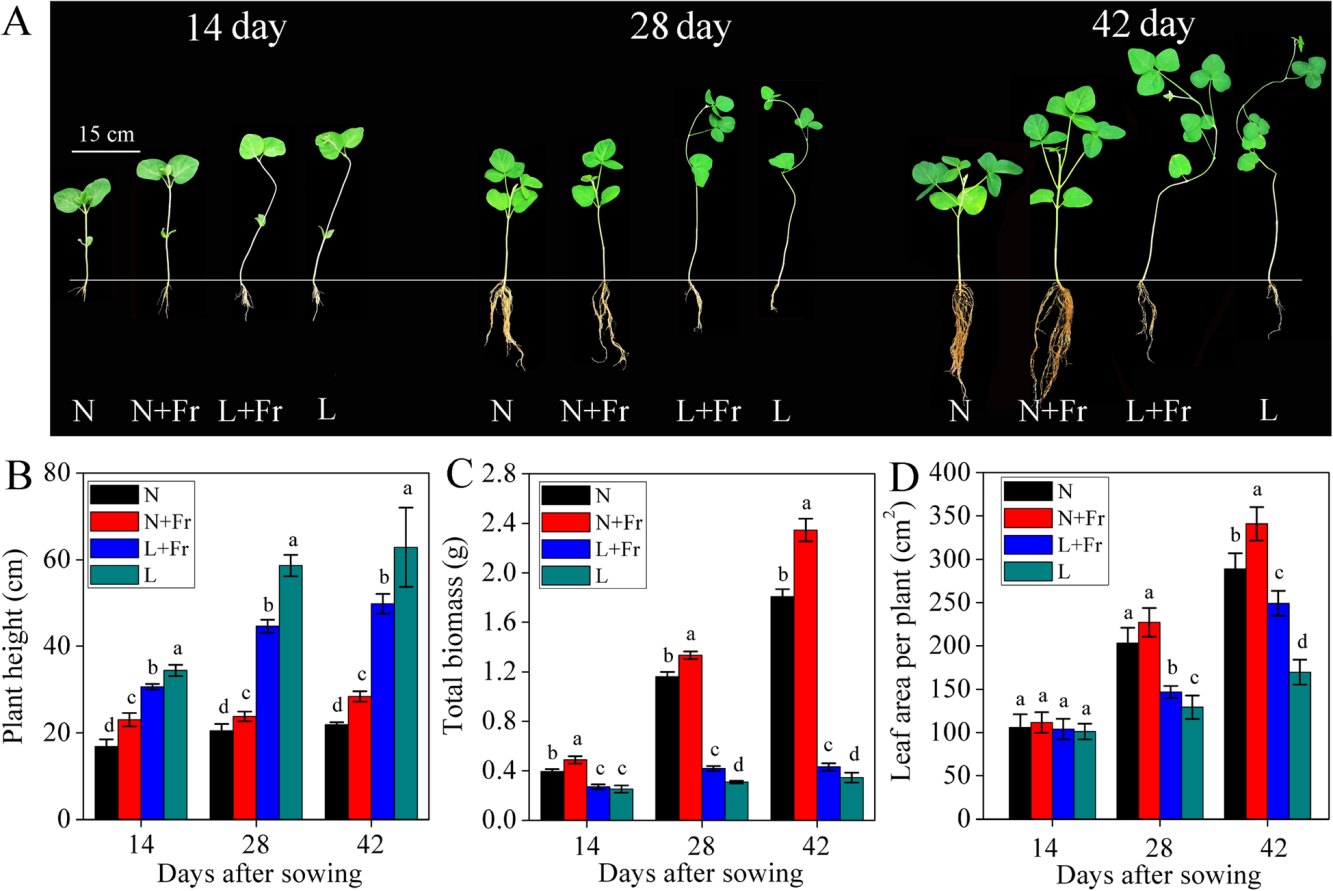
Soybean growth performance (**a**), plant height (**b**), total biomass (**c**), and leaf area (**d**) under different treatments. N, N + Fr, L + Fr, and L denote normal light (normal PAR and normal R/Fr ratio), normal light plus far-red light (normal PAR and low R/Fr ratio), low light plus far-red light (low PAR and low R/Fr ratio), and low light (low PAR and normal R/Fr ratio), respectively. Each value was expressed as the mean ± SD. The means for each treatment without common letters were significantly different at *P* = 0.05 according to Duncan’s multiple range test

Low R/Fr ratio directly affected soybean growth phenotype under normal or low light intensity (Fig. 1a). The plant height of soybean in the N + Fr treatment was significantly higher than that in the N treatment under normal light intensity. By contrast, the plant height in the L + Fr treatment was decreased by 20.8% compared with that of the L treatment in 42 days after sowing (Fig. 1b). After 14 days of sowing, the highest biomass values of soybean plants were found in treatments N + Fr and L + Fr that those in N and L. Soybean plants always produced maximum biomass in normal light treatments (N and N + Fr) as compared to those in low light treatments (L and L + Fr). At 42 days after sowing, the maximum and minimum total biomasses were 2.4 and 0.3 g plant^− 1^ in the N + Fr and L treatments, respectively. Similar trends on biomass were also found at 14 and 28 days after sowing under different treatments (Fig. 1c). In addition, low R/Fr ratio significantly increased the leaf area per plant at 14 and 28 days after sowing under normal or low light intensity, the change in the trends of leaf area per plant were consistent with the total biomass in different treatments (Fig. 1d).

### Changes in chloroplast ultrastructure, sucrose and starch content

**Fig. 2:**
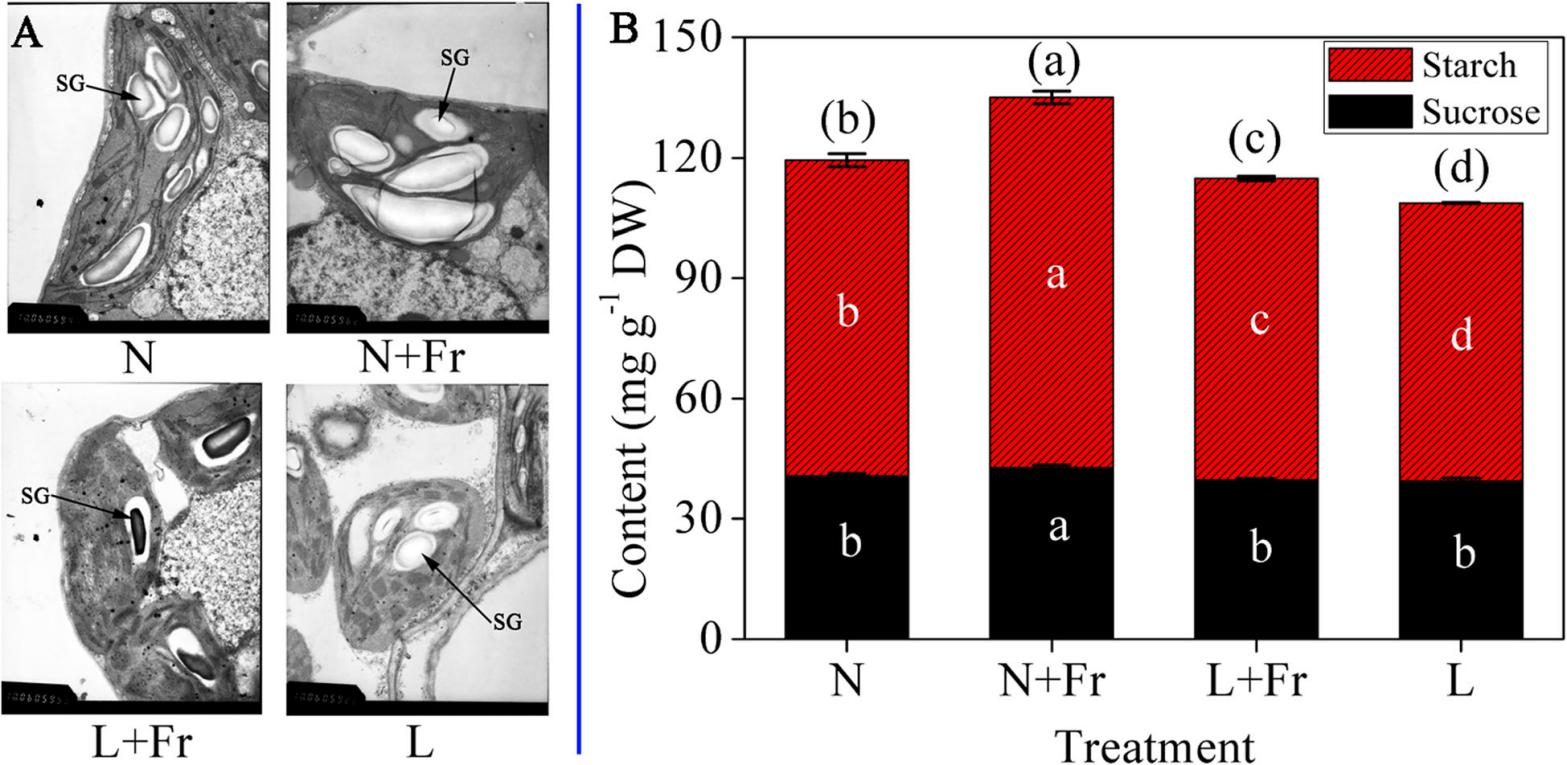
Soybean chloroplast ultrastructure (**a**), starch and sucrose contents (**b**) under different treatments. N, N + Fr, L + Fr, and L denote normal light (normal PAR and normal R/Fr ratio), normal light plus far-red light (normal PAR and low R/Fr ratio), low light plus far-red light (low PAR and low R/Fr ratio), and low light (low PAR and normal R/Fr ratio), respectively. SG stands for starch grain. Data are expressed as the means ± SD of triplicates. Means followed by different letters are significantly different at *P* = 0.05

When growing in different light environments, the changes in chloroplast ultrastructure were different, the chloroplast size in N and N + Fr treatments were larger than those in L and L + Fr treatment (Fig. 2a). Starch grain size also exhibited a similar trend. The starch contents in N + Fr and L + Fr treatments were significantly higher than those in N and L treatments, respectively. The maximum and minimum starch content were 92.4 mg/g in N + Fr treatment and 69.2 mg/g in L treatment, respectively. The sucrose content of soybean growing in N + Fr treatment was significantly higher than those in other treatments (Fig. 2b).

### Chlorophyll content, photosynthesis, and quantum yield of PS II

**Fig. 3:**
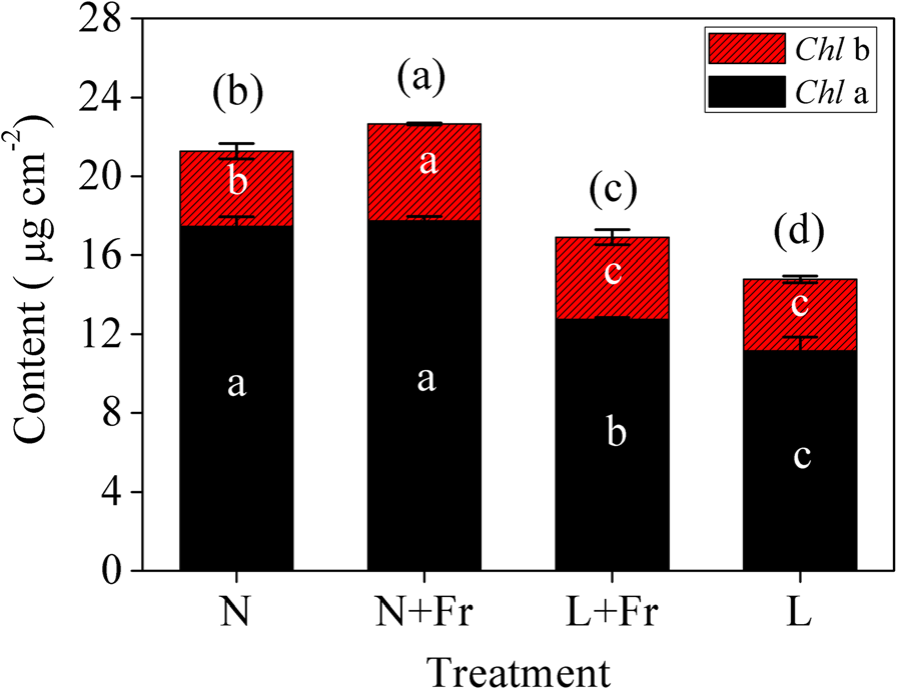
Chlorophyll (Chl) content of soybean leaves under different treatments. N, N + Fr, L + Fr, and L denote normal light (normal PAR and normal R/Fr ratio), normal light plus far-red light (normal PAR and low R/Fr ratio), low light plus far-red light (low PAR and low R/Fr ratio), and low light (low PAR and normal R/Fr ratio), respectively. Data are expressed as the means ± SD of triplicates. Means followed by different letters are significantly different at *P* = 0.05

The Chl *a*, Chl *b*, and total Chl in normal light condition (N and N + Fr treatments) were significantly lower than those in low light (L and L + Fr treatment) (Fig. 3). The total Chl contents in N + Fr and L + Fr treatments (low R/Fr ratio) were significantly increased by 6.5 and 14.3% compared with those in N and L treatments (normal R/Fr ratio), respectively.

The light response curves of the assimilation rate vs. the photosynthetic photon flux density (PPFD) and the quantum yield of PSII in four treatments were shown in Fig. S1 (data cited from our previous report [9]). Significant differences were found in the assimilation rates of the four treatments when PPFD was higher than 200 μmol m^− 2^ s^− 1^. The maximum values of photosynthetic rate (*P*_max_) and light saturation point (LSP) appeared in N + Fr treatment compared with those in other treatments. *P*_max_ and LSP under N treatment were 14.9 and 47.4% lower than the corresponding values under N + Fr treatment, respectively. Similarly, *P*_max_ and LSP under L treatment were 23.8 and 38.2% lower than those under L + Fr treatment, respectively. The minimum values of *P*_max_ and LSP appeared in L treatment compared with those in other treatments, were 5.8 μmol CO_2_ m^− 2^ s^− 1^ in the 318.2 μmol m^− 2^ s^− 1^, respectively. In this study, the quantum yields of PSII in treatments N and N + Fr was noted significantly higher than those in L and L + Fr treatments. However, treatment L + Fr enhanced the quantum yields of PSII by 15.2% as compared to those in treatment L.

### Soybean leaf proteomic analysis

**Fig. 4:**
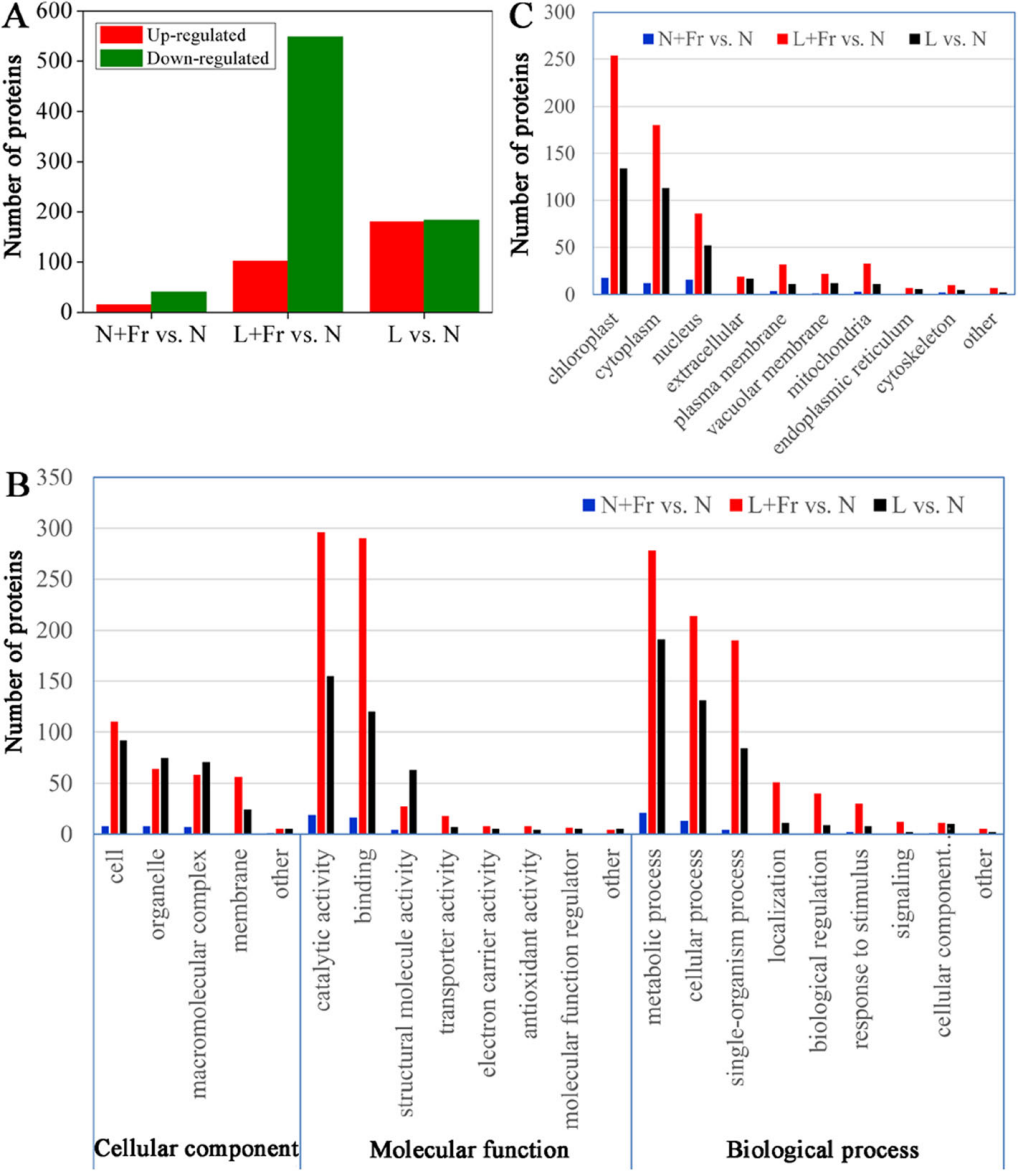
Differential protein expression analyses of soybean leaves under different light environments. **a** Histogram of the up- and down-regulated protein number under normal light plus far-red light (N + Fr), low light plus far-red light (L + Fr), and L conditions compared with those under N condition. **b** GO classification of differentially accumulated proteins. **c** The subcellular classification of the differentially accumulated protein number under N + Fr, L + Fr, and L treatments compared with that under N treatment

The total protein of the soybean leaves was extracted from different treatments, and the protein profiles were explored using the iTRAQ technique. A total of 9890 protein groups were identified, among which 7834 proteins were quantified (Table S1). On the basis of at least > 1.3- of fold change (*P* < 0.05), among the quantified proteins, we observed that 15 and 41 proteins were up-regulated and down-regulated, respectively in N + Fr vs. N; 102 and 548 were up-regulated and down-regulated, respectively under L + Fr vs. N, 180 and 183 proteins were up-regulated and down-regulated, respectively in L vs. N (Fig. 4a).

Based on Gene Ontology (GO) enrichment analysis, differentially accumulated proteins were categorized into three main groups, including biological process, molecular function, and cellular components (Fig. 4b). Biological process and molecular function categories were further divided into three sub-categories representing cellular, metabolic, and single-organism processes and binding, catalytic and structural molecule activity, respectively. The subcellular location annotation information illustrated that among all of the identified proteins, the associated chloroplast proteins accounted for 36.9, 39.1, and 32.1% of the unique proteins in L vs. N, L + Fr vs. N, and N + Fr vs. N, respectively (Fig. 4c).

**Fig. 5:**
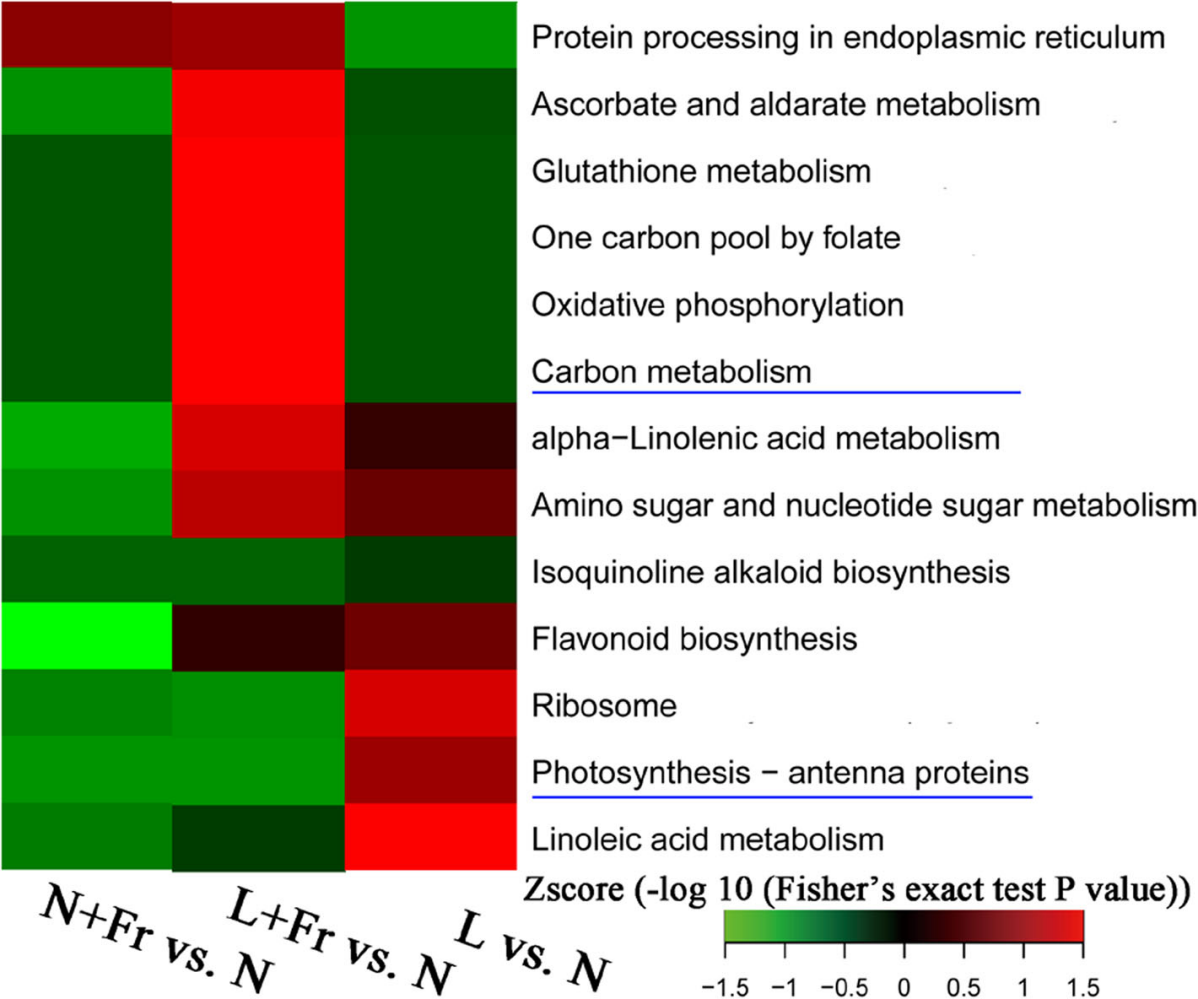
KEGG pathway-based enrichment analysis of differentially accumulated proteins. N, N + Fr, L + Fr, and L denote normal light (normal PAR and normal R/Fr ratio), normal light plus far-red light (normal PAR and low R/Fr ratio), low light plus far-red light (low PAR and low R/Fr ratio), and low light (low PAR and normal R/Fr ratio), respectively. Red colors indicate up-accumulated proteins and green colors indicate down-accumulated proteins in the N + Fr, L + Fr, and L treatments compared with the N treatment

Kyoto Encyclopedia of Genes and Genomes (KEGG) pathways visualized as heat map through a two-tailed Fisher’s exact test were used to identify the differences in protein abundance among L + Fr, N + Fr, N, and L treatments. Thirteen different functional categories were selected for analysis. As illustrated in Fig. 5, among the functional categories, C metabolism and photosynthesis-antenna proteins were related to photosynthetic CO_2_ assimilation. The proteins involved in photosynthesis in N + Fr and L + Fr treatments were down-accumulated compared with those in N treatment, whereas proteins in L treatment were up-accumulated.

### Key protein associated with photosynthesis assimilation of soybean leaves in response to different light conditions

A total of 12 differentially expressed proteins related to photosynthetic CO_2_ assimilation were detected by iTRAQ analysis under N + Fr, L + Fr, and L treatments compared with those detected in N treatment. Among these differentially expressed proteins, one protein was related to porphyrin and chlorophyll metabolism, two proteins were involved in PS I, four proteins were associated with PS II, three proteins participated in photosynthetic electron transport, and two proteins were involved in starch and sucrose metabolism (Table 1). The expression levels of nine proteins (i.e., Protochlorophyllide reductase [POR], Photosystem I subunit [PsaD], Chlorophyll a/b binding protein 1 [Lhcb 1], Lhcb 2, Lhcb 4, Lhcb 6, PetE, PetF, and Sus) were up-regulated under L treatment compared with N treatment. However, the expression levels of the two proteins (i.e., POR and Lhcb 1) in N + Fr treatment and two proteins (i.e., PsaH and PetH) in L + Fr treatment were down-regulated compared with those in N treatment.

**Table 1:** Differentially expressed proteins associated with soybean photosynthetic CO_2_ assimilation under different light environments

Accession no.	Description	N + Fr vs. N	L + Fr vs. N	L vs. N
***Porphyrin and chorophyll metabolism***				
A0A0R4J3L3	Protochlorophyllide reductase (POR)	0.75	/	2.72
***Photosystem I***				
A5Z2K3	Photosystem I subunit (PsaD)	/	/	1.38
***Photosystem II***				
A0A0R4J5I3	Chlorophyll a/b binding protein 1 (Lhcb 1)	0.37	/	3.80
Q93YG3	Chlorophyll a/b binding protein 2 (Lhcb 2)	/	/	1.52
I1JLH0	Chlorophyll a/b binding protein 4 (Lhcb 4)	/	/	1.44
I1KR46	Chlorophyll a/b binding protein 6 (Lhcb 6)	/	/	1.44
***Photosynthetic electron transport***				
C6SVR0	Plastocyanin (PetE)	/	/	1.46
C6T1J0	Ferredoxin-1 (PetF)	/	/	1.38
I1JCG8	Ferredoxin-NADP reductase (PetH)	/	0.44	/
***Starch and sucrose metabolism***				
I1MBQ9	Sucrose synthase (SS)	/	1.42	1.92
I1KAT2	Starch synthase (GlgA)	/	0.60	/

“/” indicates insignificant accumulation

### Real-time quantitative polymerase chain reaction (qRT-PCR) results confirming the differentially expressed proteins

**Fig. 6:**
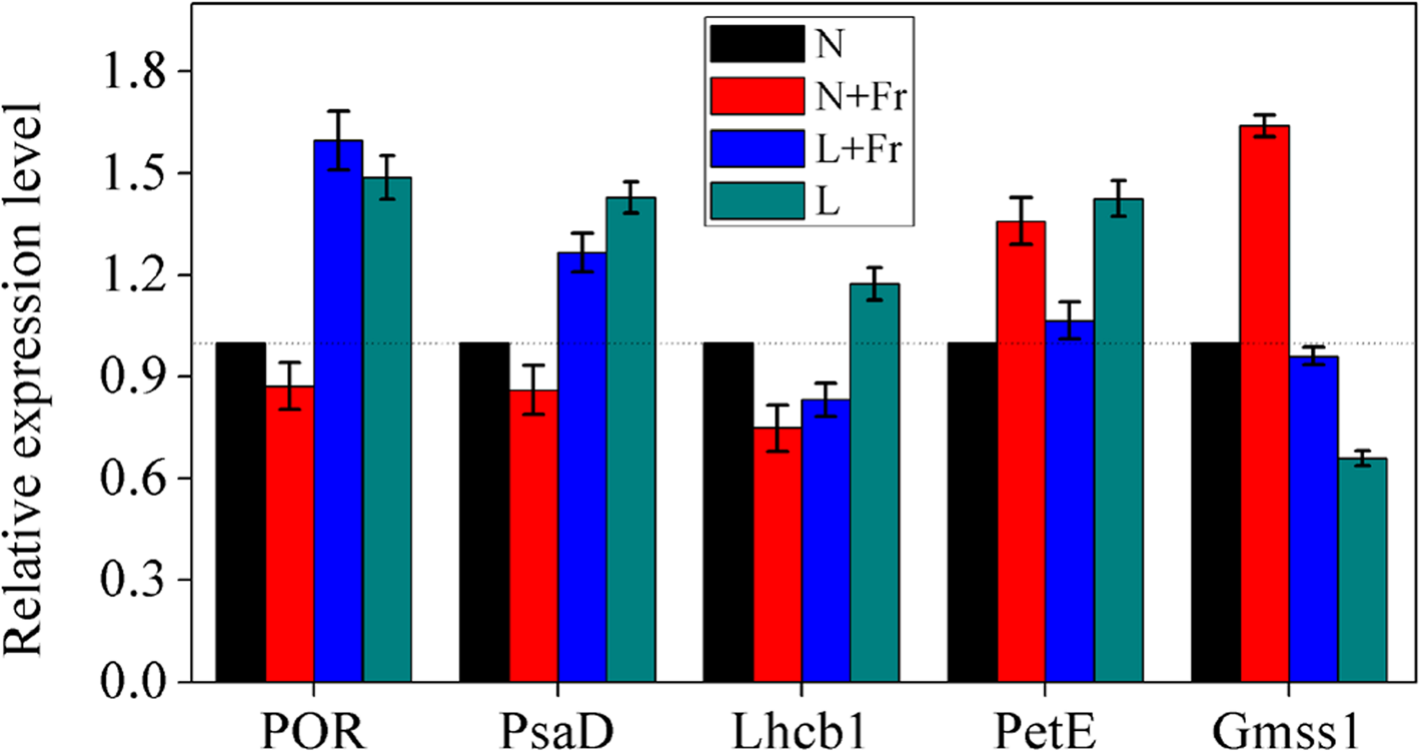
Quantitative RT- PCR validations of the genes related to the differentially expressed proteins. N, N + Fr, L + Fr, and L denote normal light (normal PAR and normal R/Fr ratio), normal light plus far-red light (normal PAR and low R/Fr ratio), low light plus far-red light (low PAR and low R/Fr ratio), and low light (low PAR and normal R/Fr ratio), respectively. POR, PsaD, Lhcb 1, and PetE represent protochlorophyllide reductase, photosystem I subunit, chlorophyll a/b binding protein 1, and plastocyanin, respectively. Values are expressed as mean ± SD (*n* = 3)

To assess the validity of the iTRAQ data, we randomly selected six gene products, including the POR, PsaD, Lhcb 1, PetE, and Sus gene expressed levels, according to differential protein classification for RT-PCR analysis, (Fig. 6). The qRT-PCR results showed that under L treatment, significant increase in the transcript level was observed for POR, PsaD, Lhcb 1, and PetE compared with N treatment. The POR, PsaD, and PetE expression levels were up-regulated under L + Fr treatment. By contrast, the expressions levels of POR, PsaD, and Lhcb 1 were down-regulated under N + Fr treatment compared with those under N treatment. The change in the Gmss 1 was different from that of POR in N + Fr, L + Fr, and L treatments.

## Discussion

A low R/Fr ratio significantly increased the stem elongation of soybean under normal light intensity, while opposite trend was found under low light intensity (Fig. 1a and b). Light intensity regulates plant height compared with R/Fr ratio under low light condition [9, 27]. However, a low R/Fr ratio significantly increased the total biomasses and leaf area of soybean under the same light intensity condition (Fig. 1c). Similarly, the whole plant biomass of geranium and snapdragon increases with additional Fr radiation (low R/Fr ratio) [6].

Photosynthesis is the basis for material accumulation. Low R/Fr ratio improved photosynthesis under normal or low light intensity condition. Similar to the reverse of Emerson enhancement effect [28], the photosynthetic efficiency of short wavelengths may be improved by increasing long wavelength. It has been observed that PSl responds to shorter wavelengths of light by exhibiting excitement compared to PSll that directly affects the photochemistry [29]. Interestingly, different wavelengths of light can supplement the effects of each other by over exiting the PSl and PSll alternatively [20]. In addition, Fr alleviates acceptor-side limitations and imposes donor-side limitations on the electron flow in PSI, and thereby increases the fraction of oxidized P700. Thus, the *P*_max_ values in N + Fr and L + Fr treatments (high enrichment of Fr light) were significantly higher than those in N and L treatments, respectively. Although low light intensity significantly decreased the Chl content (Chl *a* and *b*) compared with normal light condition, a low R/Fr ratio significantly increased the Chl content per unit area under the same light level [16]. The changes in the trends of leaf area, Chl content, and *P*_*n*_ were consistent with the total biomass in different treatments (Figs. 1, 3, S1), thereby indicating that low R/Fr ratio might improve the photosynthetic capacity of soybean in the same light intensity.

In this study, a low R/Fr ratio under N + Fr and L + Fr treatments can significantly increase the starch and sucrose content with respect to the corresponding values under N and L treatments, respectively (Fig. 2b). The complementation of shorter wavelengths of light with far-red light is responsible for the establishment of an excitation balance between two photosystems. This excitation balancing might optimize the rate of photochemistry and relative CO_2_ assimilation [20]. Similarly, the change in trends of starch and sucrose content in different treatments were similar to *P*_*n*_, quantum yield of PSII, and total biomass (Figs. 1 and 2) [9]. In addition, the promotive effect of additional Fr radiation on photosynthesis and whole-plant net assimilation was previously reported in lettuce and geranium [12, 18]. Together, these results suggested that the high enrichment of Fr light possibly contributed to increased starch and sucrose contents by improving photosynthetic capacity of soybean leaves.

Proteomics is an approach for the systematic identification of all proteins expressed in a cell or tissue [30]. In this study, we mainly focused on the differentially expressed proteins related to photosynthesis (Table 1). The POR is the key functional protein in regulating chlorophyll metabolism [29, 31]. The POR protein expression was up-regulated under the L treatment relative to that under the N treatment, whereas an opposite trend was found in N + Fr treatment (Table 1). POR expression significantly increased with the decrease in irradiance [32]. Our previous research also found that maize shade or low light can improve the POR protein expression [11]. The qRT-PCR analysis results confirmed the observed response of POR to different light intensities and qualities (Fig. 6). POR is a negative control protein of light-dependent response, especially for light intensity [33]. Although low R/Fr ratio in the same light intensity condition can improve the Chl contents per unit leaf area (Fig. 3), opposite trends were found in the POR protein and gene expression levels (Table 1; Fig. 6). This result was related to the unit (mg cm^− 2^) of Chl contents [11, 34].

The PsaD subunit of PS I is a peripheral protein that provides a docking site for ferredoxin and interacts with other PS I subunits [35]. PsaD is highly sensitive to the light environment, and high light intensity significantly decreases the PsaD content [36]. Similarly, low light up-regulated the PsaD protein and gene expression levels because the high protein expression of PS II can improve the electron transport, which increases the PsaD protein expressions under low light condition [11]. The low R/Fr ratio (Fr light enhancement) may reverse the PsaD protein expression under low light condition (Table 2). This finding was similar to the results showing Fr light can improve the efficient photochemistry and photosynthesis [18].

**Table 2:** Light intensity and red/ far-red ratio of soybean canopy in different treatments

Treatment	N	N + Fr	L + Fr	L
PAR (μmol·m^−2^·s^−1^)	566.50 ± 2.24a	566.57 ± 1.84a	64.22 ± 0.68b	63.33 ± 0.14b
R/Fr ratio	1.33 ± 0.081a	0.42 ± 0.05b	0.08 ± 0.012c	1.26 ± 0.027a

Different letters in each table row are significantly different at *P* = 0.05

The light-harvesting antenna (LHCII) is the major pigment- protein complex associated with PS II, thereby forming the PS II-LHCII supercomplex in which several LHCIIs surround the reaction center core complex [37]. The Lhcb levels decrease as light intensity increase with a significant trend for Lhcb 1, Lhcb 2, and Lhcb 4 [5, 6, 37]. Our results agreed with these data (Table 1). Similarly, the protein Lhcb 1 and Lhcb 2 expression levels decreased at excessive irradiance [38]. However, Lhcb 1, Lhcb 2, Lhcb 4, and Lhcb 6 levels remained unchanged under L + Fr treatment (low R/Fr ratio) compared with those under N treatment. Similar result also was found in the gene expression of Lhcb 1 (Fig. 6). These results indicated that increasing the Fr light wavelength (low R/Fr ratio) can improve the photosynthetic efficiency under short wavelength. Ahmadova and Mamedov [39] indicated that the low energy of the Fr photons plays an important role in the photochemical progress.

PetE is one of the low molecular weight proteins which is involved in the cyclic and linear electron transport in oxygenic photosynthetic organisms. It catalyzes the electron transfer from the membrane-bound Cyt b6/f complex to P700 [40]. PetH is a redox partner protein of PetF, which is a light-dependent electron transfer protein [41]. These proteins are highly sensitive to the light environment. Low light condition can up-regulate PetE and PetF expression (Table 1, Fig. 6). The changes in PetE and PetF protein trends were similar to those of Lhcb 1, Lhcb 2, Lhcb 4, and Lhcb 6 proteins under low light condition. This phenomenon may have occurred because the degree of increased thylakoid stacking improved the light capture and electron transport by up-regulating relative proteins in low light compared with those in normal light (Table 1) [42]. However, the low R/Fr ratio (N + Fr and L + Fr treatments) barely affected the PetE and PetF expression level compared with normal R/Fr ratio (normal light). This result was similar to the findings of our previous research [11]. The change in PetH expression was different from that of PetF under different treatments (Table 1), because PetH is rate-controlling protein in the protein–protein interaction reaction between PetH and PetF as the electron transfer partner (Okada, 2009). This finding may explain why the chlorophyll a/b binding proteins up-regulated in low light but *P*_*n*_ decreased compared with other treatments (Fig. S1).

Leaf starch and sucrose metabolism progress was also affected by light environment (Fig. 2) [43]. The SS expression levels were significantly up-regulated under L and L + Fr treatment (Table 1). However, an opposite trend was found in the gene expression pattern of Gmss1 under the L and L + Fr treatment (Fig. 6). Similarly, the sucrose level in low light condition decreased first due to decrease in the activities of sucrose synthesizing enzymes (i.e., SPS and SS-s) [44]. These results may be related to the different gene and protein expression levels for the same protein under different treatments.

## Conclusion

These results suggested that low R/Fr ratio (high enrichment of Fr light) in the same light intensity can increase the photosynthetic CO_2_ assimilation by improving the photosynthetic capacity.

## Methods

### Plant materials and treatment design

Nandou 12, which is a major soybean cultivar that is closely planted or intercropped with other crops in agriculture production in Southwestern China, was selected as the experimental material. Soybean seeds were provided by the Nanchong Institute of Agricultural Sciences, Sichuan Province, China. All soybean seeds were covered with wet filter paper for 24 h at 30 °C. After that, the germinated soybean seeds were grown in containers with a seedling spacing of 10 cm. The size of each container was 15 cm in height, 40 cm in length, and 20 cm in width, and filled with humidified organic soil.

The experiment was divided into two parts. One part was used for morphology measurement. The containers including germinated seeds were directly placed under different light environment treatments, and each treatment included three containers.

The other part was used for physiological and proteomic analyses. The difference in soybean growth period under the various light environments was avoided by growing soybean under normal ambient condition, until the complete development of the first trifoliate leaf. Then, plants were subjected to four different light treatments just after the appearance of the second trifoliate. Furthermore, after 15 days of treatments, all samples were collected at 10 am for the analysis of physiological parameters and differentially expressed proteins. Plants were grown in growth chamber with natural solar radiation, with the temperature maintained at 25 °Cin the day and 20 °C during the night. Additionally, the humidity was maintained at 60%, and plants were irrigated with nutrients solution (0.2% Hoagland’s solution) after every couple of days [45].

The black nylon gauze and Far Red (Fr)-LED was used according to our previous knowledge [9], to control the PAR and Red-Far Red ration in soybean canopy. The intensity of Fr light in soybean canopy is 5.98 ± 0.14 μmol·m^− 2^·s^− 1^ under dark condition. The PAR and spectral irradiance of soybean canopy were measured at noon on a sunny day, every measurement was replicated five times. The following four treatments were used (Table 2): normal light, normal light plus Fr light, low light, and low light plus far-red light. Every treatment was repeated three times in three different growth chambers. The PAR and spectral irradiance were measured using LI-190SA quantum sensors (LI-COR Inc., Lincoln, NE, USA) and a fiber-optic spectrometer (AvaSpec-2048; Avantes, Netherlands) placed at 10 cm above the soybean canopy, respectively [7, 9].

### Morphological characteristics

The plant height from the soil surface to the growing point of soybean. The biomass and leaf area of five soybean seedlings were measured every 14 days after 14 days of sowing under four treatments. Leaves were scanned using a flatbed scanner (CanoScan LiDE 200, Canon Inc., Japan), and the leaf area (cm^2^) was measured by Image J 1.45 s. Biomass samples were over-dried at 105 °C for 0.5 h to stop metabolic processes of tissues and then dried at 80 °C for 72 h to a constant weight [9].

### Chloroplast ultrastructure

As previously published by Yang et al. [11], the leaf segments (2 mm × 2 mm) of second trifoliate were placed at 4 °C in 3% glutaraldehyde, and treated with 1% osmium tetroxide. Then, the fixed segments of soybean leaves were dehydrated and embedded in a graded acetone series and Epon812, respectively. The semithin sections were stained and cut with a diamond knife. Then, the samples were stained with acetate and lead citrate, and observed using a transmission electron microscope (TEM; HITACHI, H-600IV, Japan).

### Measurements of sucrose and starch

Leaf samples were over- dried at 105 °C for 0.5 h to stop metabolic processes of the tissues and then dried at 80 °C for 24 h to a constant weight. According to the methods of Lee et al. (2020) with some changes [45], soluble sugars were extracted from 0.5 g dried samples by homogenization in 5 ml of 80% (v/v) ethanol. After heating the homogenate in a water bath, the insoluble fraction was removed by centrifugation at 3500 g for 10 min. The precipitate was homogenized and centrifuged again. Supernatants were pooled and then diluted up to 25 ml with 80% ethanol. The hydrolyzed samples were subjected to centrifugation, and only a small amount (100 uL) of supernatant was collected and added with 100 uL of 30% Potassium Hydroxide solution followed by boiling, about 10 min. The solution was cooled down, and the anthrone agent was added. After heating the solution again at 40° for 15 min, it allowed to cool, and absorbance was checked at 620 nm. The amount was calculated using the standard solutions of sucrose. The leftover material is centrifuged tube was utilized for starch extraction, by adding 2 mL of water. Then, the tubes were placed in boiling water bath for 15 min. After cooling, 2 ml of 9.2 M perchloric acid (PCA) was added. After stirring for 15 min, the supernatants were collected after centrifuging the contents at 3500 g for 10 min. The residues were re-extracted two times with 2 ml of 4.6 M PCA. After centrifugation, the supernatants were combined, volumes were made to 50 ml with water. Starch was determined colorimetrically using the phenol-sulphuric acid method, as described by [46].

### Photosynthesis, photosynthetic pigment concentration, and quantum yield of PS II

As described by Yang et al. [2], the second trifoliolate leaf was selected to measure photosynthetic characteristics using a Li-6400 portable photosynthesis system (LI-COR Inc., Lincoln, NE, USA), environment temperature 25 °C and a CO_2_ concentration of 400 μmol mol^− 1^ from 9:00 to 11:00. Eleven light intensity levels (0, 20, 50, 100, 150, 200, 400, 600, 800, 1000, and 1200 μmol m^− 2^ s^− 1^) were imposed. On a light response curve, PPFD was located on the horizontal axis and *P*_*n*_ was on the vertical axis (*P*_n_-PPFD curve). The *P*_max_ and LSP were then estimated using the method proposed by Yang et al. [11]. Chlorophyll fluorescence parameters were obtained using a CI Imager chlorophyll fluorescence imaging system (Technologica Ltd., Colchester, UK). Before each measurement, we placed soybean leaves of each treatment under dark conditions for 10 min. Then actinic illumination (750 μmol m^− 2^ s^− 1^) was switched on, and saturating pulses were applied at 20 s intervals for 15 min. From each of these, the maximum fluorescence (*F′*_m_) and the steady-state fluorescence (*F*_s_) were determined in the light condition. The quantum efficiency of the photosystem II was calculated according to the formula (*F′*_m_- *F*_s_)/ *F′*_m_ [9].

After that, four-leaf disks of 15 mm diameter were obtained from the center of each leaf and then cut into pieces of 3 mm. These leaf-disks were placed in 10 ml of 80% acetone in the dark at 20 °C for 24 h. The chlorophyll contents were determined by following a previously published procedure [34]. Triplicates were prepared for each treatment.

### Protein extraction, digestion, and iTRAQ labeling

After 2 weeks of treatment, the second leaf of soybean plants was ground using liquid nitrogen. Following the method of Yang et al. [11], the powder then shifted to a centrifuge tube. It fragmented using lysis buffer and 1% protease suppressor cocktail under the cold condition with the help of a high-intensity ultrasonic processor (Scientz). The leftover was preceded by centrifugation. Proteins were sedimented under cold using 15% TCA for 2 h at − 20 °C. Following centrifugation at 4 °C, the afloat was disposed of, and the leftover sediment was rinsed three times using cold acetone. The protein concentration was then determined by dissolving the protein in the buffer. The reduction and alkylation of protein solution were done for 45 min using 20 mM IAA at room temperature in the dark. 100 mM TEAB was then used for the dilution of the protein sample. At last, for the digestion of protein, trypsin was added using the trypsin-to-protein ratio. Approximately 100 μg of protein was digested with trypsin in each sample for further experiments. For iTRAQ labeling, peptides were desalinized with the help of Strata X C18 SPE column (Phenomenex), vacuum-dried, replenished in 0.5 M TEAB, and then operated using 4-plex iTRAQ kit manual instructions.

### HPLC fractionation and LC-tandem mass spectrometry (MS/MS) analysis

As previously reported [11], high PH reverse-phase HPLC divided the sample into fractions by using Agilent 300 Extend C18 column. Following the dehydration through vacuum centrifugation, the peptides were dissolved in 0.1% Formic Acid and loaded onto a reverse-phase analytical column (Acclaim PepMap RSLC, Thermo Scientific). EASY-nLC 1000 UPLC system was used to perform Gradient elution at a constant column flow rate of 350 nl/min. The resulting peptides were then analyzed using an Orbitrap Fusion™ Tribrid™ mass spectrometer (Thermo Fisher Scientific).

### Database search and analysis

MaxQuant, with an integrated Andromeda search engine (v.1.5.2.8), was used to process the MS/MS data. iTRAQ 8-plex was used for the quantification, and the default values of all the other parameters in MaxQuant were selected, as reported by Yang et al. [11]. To identify the down-regulated or up-regulated protein expression, we used 0.77- or 1.3-fold cut-off with a *P* < 0.05, respectively. GO annotation was used to annotate the proteins [23]. The differentially accumulated proteins were also assigned to the KEGG database [47, 48].

### qRT-PCR verification

Proteomics reliability was confirmed by the help of qRT-PCR assay. RNA isolation was done according to the protocol of Yuan et al. [49]. In our study, the β-tubulin gene was taken as a reference control. RT-PCR was done on a CFX96 system machine (Bio-Rad, USA). The primers used are listed in the Table S2.

### Statistical analysis

Significance was calculated using one-way ANOVA by computer-based program SPSS (version 16.0). Data are given as mean ± standard deviation from three replicates. Statistical significance was recognized at *P* < 0.05.

## Supplementary information

**Additional file 1: Figure S1.** Light response curves of net photosynthetic rate (A), light saturation point (B), and quantum yield of PSII (C) of soybean leaves under different treatments. N, N + Fr, L + Fr, and L denote normal light (normal PAR and normal R/Fr ratio), normal light plus far-red light (normal PAR and low R/Fr ratio), low light plus far-red light (low PAR and low R/Fr ratio), and low light (low PAR and normal R/Fr ratio), respectively. *P*_*max*_ and LSP represent the maximum photosynthetic rate and the light saturation point. Data are expressed as the means ± SD of triplicates. Means followed by different letters are significantly different at *P* = 0.05.

**Additional file 2: Table S1**. Differentially expressed statistics of unique proteins.

**Additional file 3: Table S2**. List of primers for characterizing *Glycine max* genes.

## Data Availability

The datasets used and analysed in this study are available from the corresponding author.

## Abbreviations

Chl: Chlorophyll
GO: Gene Ontology
iTRAQ: Isobaric tags for relative and absolute quantification
KEEG: Kyoto Encyclopedia of Genes and Genomes
LSP: Light saturation point
*P*_n_: Net photosynthesis rate
*P*_max_: Maximum values of photosynthetic rate
qRT-PCR: Real-time quantitative polymerase chain reaction
R/Fr: Red /Far-red ratio
PAR: Photosynthetically active radiation
POR: Protochlorophyllide reductase
PPFD: Photosynthetic photon flux density
PS I: Photosystem I
PS II: Photosystem II
