# Supplementary material for: Low red/far-red ratio as a signal promotes carbon assimilation of soybean seedlings by increasing the photosynthetic capacity
Source: BMC Plant Biol. 2020 Apr 8;20:148. doi: 10.1186/s12870-020-02352-0 (PMC7140557; doi:10.1186/s12870-020-02352-0)
Supplement: Supplementary file 1 — Additional file 1: Figure S1. Light response curves of net photosynthetic rate (A), light saturation point (B), and quantum yield of PSII (C) of soybean leaves under different treatments. N, N + Fr, L + Fr, and L denote normal light (normal PAR and normal R/Fr ratio), normal light plus far-red light (normal PAR and low R/Fr ratio), low light plus far-red light (low PAR and low R/Fr ratio), and low light (low PAR and normal R/Fr ratio), respectively. Pmax and LSP represent the maximum photosynthetic rate and the light saturation point. Data are expressed as the means ± SD of triplicates. Means followed by different letters are significantly different at P = 0.05. [file 12870_2020_2352_MOESM1_ESM.docx]

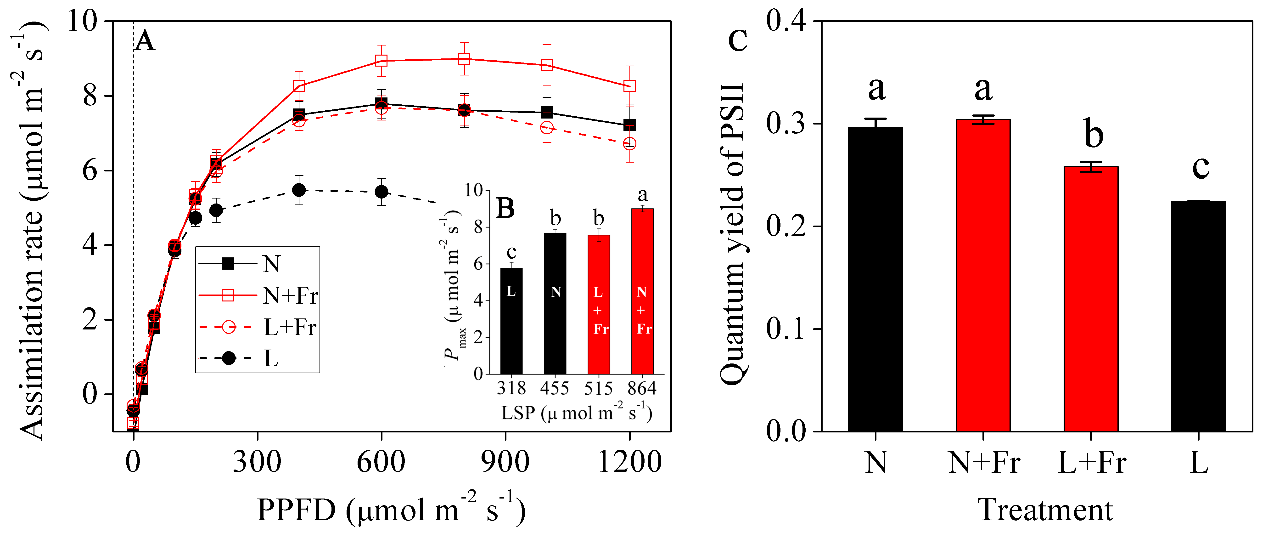


**Figure S1.** Light response curves of net photosynthetic rate (A), light saturation point (B), and quantum yield of PSII (C) of soybean leaves under different treatments (data cited from our previous report [9]). N, N+Fr, L+Fr, and L denote normal light (normal PAR and normal R/Fr ratio), normal light plus far-red light (normal PAR and low R/Fr ratio), low light plus far-red light (low PAR and low R/Fr ratio), and low light (low PAR and normal R/Fr ratio), respectively. *P_max_* and LSP represent the maximum photosynthetic rate and the light saturation point. Data are expressed as the means ± SD of triplicates. Means followed by different letters are significantly different at P=0.05.
