# Supplementary material for: Low red/far-red ratio as a signal promotes carbon assimilation of soybean seedlings by increasing the photosynthetic capacity
Source: BMC Plant Biol. 2020 Apr 8;20:148. doi: 10.1186/s12870-020-02352-0 (PMC7140557; doi:10.1186/s12870-020-02352-0)
Supplement: Supplementary file 3 — Additional file 3: Table S2. List of primers for characterizing Glycine max genes. [file 12870_2020_2352_MOESM3_ESM.docx]

**Table S2.** List of primers for characterizing Glycine max genes

| **Gene Location （Annotation）** | **Protein**  **Location** | **Forward primer** | **Reverse primer** |
| --- | --- | --- | --- |
| *LOC100813807* | A0A0R4J3L3 | TGGCCTGCCGGGATTT | CAATGCCGGCAGATTTCG |
| *GmpsaD* | A5Z2K3 | GGTGGAGGAGTTTTATGTCATTACG | CCAGTGGGCATTTCAAAGATC |
| *LOC100799813* | A0A0R4J5I3 | CTGGTGAGCCCCCATCCTA | CAGCAGTGTCCCAACCATAGTC |
| *LOC100499708* | C6SVR0 | TGCCGCCGCAGTTAAAGT | CCTTGATGCCGCAGAACCT |
| *Glyma.13G114000* | *Gmss1* | GGCATCCTGCAACACCAC | GAACAGCCAGAGCAACCC |
| *β-tublin* | - | CCTTCCTTGGCAACTCGACA | TCGTCCATACCCTCCTGTGT |
